# LEAVENED APPREHENSIONS: BREAD SUBSIDIES AND MORAL ECONOMIES IN HASHEMITE JORDAN

**DOI:** 10.1017/S0020743818000016

**Published:** 2018-05-10

**Authors:** José Ciro Martínez

**Affiliations:** PhD Candidate in the Department of Politics and International Studies, University of Cambridge, Cambridge, UK

**Keywords:** anthropology, food, hegemony, moral economies, politics of provision

## Abstract

This article analyzes the microprocesses that imbue bread with meaning and the macropolitics that shape its subsidized provision. It begins by outlining bread’s multiple forms of value and significance, some easily quantifiable, others not. It problematizes the predominant approach to studying moral economies before putting forth an alternative framework. Drawing on eighteen months of fieldwork in Jordan, the following empirical sections examine the different ways in which bureaucrats, bakers, and ordinary citizens portray the government’s universal subsidy of Arabic bread. I unpack the diverse opinions encountered in the field and discuss their links to the Hashemite regime’s polyvalent legitimating discourse. The article then dissects the politics of provisions that contribute to the bread subsidy’s paradoxical persistence. It concludes by considering the relationship between moral economies, opposition politics, and authoritarian power in the context of Jordan’s ongoing food subsidy debate.

Every morning Saʿid wakes up, hastily puts on a pair of slippers and track bottoms, and sets out on a three-block stroll to al-Hayat, a bakery owned by Hamdi Barjus. The establishment is located in Jabal al-Nadhif, a densely populated neighborhood in Amman’s poorer eastern quarter. Purchasing bread is Saʿid’s one daily task, the chief responsibility of the currently unemployed member of the Taʿimi family. On one of our many walks together to the bakery, I asked Saʿid why he is entrusted with this assignment. “I am the youngest of my brothers and the only unemployed one. The others are preparing for work or readying their kids for school,” he told me. “And bread is cheap, they know I will always have enough money to buy what we need.” I wondered in jest about what would happen if we did not arrive with the always-anticipated three kilograms of bread. “Big trouble, I think. How else would we have breakfast? The bread feeds four families, my two single brothers as well as any other guests.” “And is the bread always available?” I asked naively, “Who makes sure there is enough?” “Barjus is an excellent businessman. He has the production line down to a science,” he answered. “But I suppose it is the state’s responsibility. When there is no bread people cannot eat, they get angry.”

Jabal al-Nadhif suffers from high levels of poverty and unemployment. With the acceleration of neoliberal economic reforms and the concomitant decline in the government’s redistributive expenditures since 1999, most welfare services in Nadhif and other poor neighborhoods in East Amman have been left to NGOs, charitable arms of political groups, and religious or community organizations.^1^ These bodies have replaced government-run welfare programs as the main source of mediation between individuals, communities, and the vagaries of the market. In Nadhif, the lack of public investment is unmistakable.^2^ The neighborhood’s streets are dirtier than the tidy avenues of wealthier Abdun, its public schools lie in tatters, and police are rarely seen. Yet every day, five perpetually bustling bakeries selling subsidized bread serve the neighborhood’s low-income residents. Why, amid extensive reductions in other social welfare expenditures, have changes to this one program elicited widespread angst and opposition? Bread is different, many told me. Its dearth causes anger and unrest; its subsidized provision fights hunger while fostering social stability. Although at various points the Jordanian government has subsidized a host of basic foodstuffs (frozen poultry, milk powder, rice, cooking oil) and other household items (tea, fuel, water), the vast majority of these undertakings have been scaled back, if not completely eliminated. Nevertheless, the bread subsidy remains.

Over time, the accessibility and availability of bread has become closely linked to a perceived right to subsistence among Jordanian citizens. The foodstuff’s provision is also frequently described as a sacrosanct responsibility of the state. During the most recent bread subsidy debate (2013–15), one prominent journalist referred to the welfare program as “the last red line” in the dwindling social pact between rulers and ruled.^3^ Similarly, influential MP Assaf al-Shubaki declared in a meeting of parliamentarians that, “the loaf of bread is a red line . . . it is a source of dignity among Jordanians who will not accept a rise in its price.”^4^ For Sulayman al-Shiyab, head of the Islamic Action Front’s Economic Council, “Bread is not just food. When it hits the ground we pick it up, press it to our forehead and kiss it. Bread represents justice, dignity, life itself. *Al-khubz nʿama* [bread is a blessing from God].”^5^ In response to popular apprehensions and disapproving remarks expressed amid the first rumors of a potential bread price hike in early 2013, Prime Minister Abdullah Ensour repeatedly affirmed: “Bread [prices] will not increase 1 *fils* [penny] for citizens.”^6^ Ensour’s precautionary statements are hardly a manifestation of unwarranted paranoia. During the last twenty-five years, many of the most significant acts of protest in the country have been triggered by the government’s retraction of subsidies and other consumer protections.^7^ These previous incidents, as well as the more mundane instances of angst and disapproval witnessed during Jordan’s recent food subsidy debate, illustrate the contested nature of welfare systems. They also point to the multifaceted importance of bread in the Hashemite Kingdom of Jordan.

Why is this foodstuff imbued with meanings beyond its nutritional value? How do these relate to political dynamics and policy outcomes? To answer these questions, this article dissects moral economies of bread amongst a diverse set of Jordanian citizens. Anchoring them in a framework reliant upon Antonio Gramsci’s understanding of hegemony, it examines the politics of provisions that undergird the foodstuff’s subsidy. The analysis is informed by semistructured and informal interviews with government officials and bakery owners (n=128). It also draws on participant observation undertaken during eighteen months of ethnographic research between 2013 and 2015, which included twelve months working in three bakeries in different socio-economic milieus in the Jordanian capital of Amman. Pseudonyms are used and bakery names changed when requested or deemed necessary for anonymity; all referenced interviews were conducted on the record.

The article begins by outlining bread’s multiple forms of value and significance in Jordan, some easily quantifiable, others not. It problematizes predominant approaches to studying moral economies before putting forth an alternative framework to analyze the views articulated by producers, consumers and distributors when discussing bread. In this section, I will draw on Lara Deeb and Mona Harb’s concept of moral rubrics, defined as the “different sets of ideas and values that are revealed and produced through discourses and actions,” while acknowledging the multiple positions that individuals and communities occupy and the diverse sets of power relations they encounter.^8^ This combination allows me to trace how the provision of a specific foodstuff relates to unequal relationships of exchange shaped in part by moralities of reciprocity, mutual obligation, and protection.^9^ The article then summarizes the history of Jordan’s bread subsidy to contextualize how the welfare program became rooted in popular expectations and public policy. The following three sections examine the different ways in which bureaucrats, bakers, and ordinary citizens portray bread and its subsidy. The final section relates these moral economies of bread to the politics of provisions that shape welfare outcomes in the Hashemite Kingdom. Through close scrutiny of bread politics, this article seeks to shed light on the fragmentary yet persistent nature of authoritarian power(s), as well as the forms of resistance, adaptation, and acquiescence that characterize Jordanian neoliberalization. Above all, it seeks to make clear the importance of normative alignments and moral economies to systems of social provision.

## WHY BREAD?

Bread’s nutritional importance is primarily tied to its centrality in the diets of Jordan’s poor. In 2014, residents of the country were estimated to consume nearly ten million loafs of *khubz* ʿ*arabī* (Arabic—or pita—bread) a day, averaging around ninety kilograms of bread per person annually.^10^ Reliance on subsidized bread is most pronounced in Jordan’s poverty pockets, where average monthly food expenditures are dedicated almost entirely to cereals.^11^ Yet support for this welfare program is not limited to the Jordanian poor. Cheap bread functions as an emergency relief program for Syrian refugees.^12^ It also helps feed migrant and salaried workers, effectively subsidizing the labor costs of small and large businesses. Lastly, the bread subsidy helps Jordan’s middle class maintain a certain quality of life, which helps explain why support for the policy defies a simple class logic.

Nevertheless, bread’s importance is not only a product of its nutritional or financial properties. Due to their ties to health, customs, and communal relations, certain foods become omnipresent elements in everyday life. They lie at the heart of social identities and can function as an articulation of entrenched traditions and cultural idioms that develop over time.^13^ One way this occurs is through commensality. The act of eating together is a widespread routine practice, one through which people can become bound together, slowly transforming a group into “we.”^14^ Across Jordan, the act of eating bread collectively fosters relations and obligations, ties expressed to me in phrases such as *baynuna¯ khubz wa-meleh_._*(there is bread and salt between us). This commensality can also foster fleeting feelings of shared experience with others. As Jane Cowan argues for dance in the context of northern Greece, eating can

provoke that sense of recognition—which though not inevitable is still by no means rare—that one is morally part of, just as one is now corporeally merged with, a larger collectivity, a recognition that, as a profoundly visceral knowledge, carries the force of absolute conviction.^15^

Staple foods such as bread take on their meanings partly through such patterns of communal consumption among members of a social group, in the process obtaining metaphoric associations with collective values and coveted circumstances.

In the vast majority of interviews and informal conversations that I had with bakers and ordinary citizens, the availability of discounted *khubz* ʿ*arabī* was repeatedly associated with values such as *ada¯ la* (justice), *al-tad_._a¯ mun al-ijtima¯ ¯* ʿ*ı* (social solidarity), and *al-taka¯ful al-ijtima¯* ʿ*ī* (mutual responsibility). Bread’s role as a subsistence good coupled with its cultural symbolism generates meanings that work in turn to make “the material and the symbolic indistinguishable.”^16^ The foodstuff’s status as a common culinary denominator among the diverse constituents of the continuously (re)imagined Jordanian nation means it transcends its physiological functions; bread is a communal social good that links individuals as citizens.^17^ *Khubz* ʿ*arabī*’s availability is not solely prized as a physical foundation of livelihood, which is why its importance cannot be fully captured by quantitative measures, the “visual representations of order and efficiency” that overlook alternative systems of value.^18^ Furthermore, the meanings with which bread has been imbued—the ideas, beliefs, and values that contribute to its significance—are politically salient. Debates over social policy and distributive justice, and not only in the MENA region, are swayed by arguments that draw on *a¯khla¯q* (morality),^19^ a socially constructed set of equivocal standards and judgments against which behaviors are assessed, the “forms and acts by which commitments are engaged and virtue accomplished . . . albeit in the face of numerous constraints.”^20^ So long as the ruling order and the citizenry justify their actions with reference to values and morality, the struggle for the symbolic high ground remains crucial.^21^

## MORAL RUBRICS AND MORAL ECONOMIES OF BREAD

During Jordan’s most recent subsidy debate (2013–15), consumers, producers, and distributors of bread repeatedly attributed nonquantifiable meanings to the foodstuff. Many citizens viewed its circulation to be linked to certain values, which were emphasized when they articulated their policy preferences. These sets of normative commitments were produced and revealed through discourses and practices that took place in religious, civic, and political registers.^22^ What is interesting, and different, from most accounts of past and present moral economies is how varied and internally diverse the values underpinning public claims were. Evidently, no one single value or cultural logic underpins morality. The latter is constantly redefined in relation to flexible rubrics in which certain ideals and values emerge as primary.^23^ These moral rubrics are shaped by everyday negotiations between different and sometimes-competing sets of norms that are consistently questioned and reinterpreted in the process of arriving at definitions of the good, the just, and the desirable. They lie at the heart of moral economies, which are relational but never static, specific to each polity and person and constantly being defined anew.^24^

Although this article’s focus on access to subsistence goods more closely mirrors the empirical concerns of E.P. Thompson, the conception of moral economy it employs emerged in conversation with the work of James Scott and his interlocutors.^25^ In *The Moral Economy of the Peasant*, Scott dissects the interplay between cultural values and perceptions of exploitation related to “subsistence security” among the Vietnamese and Burmese peasantry.^26^ He calls the product of this interplay a “moral economy,” defined as the subaltern classes’ “notion of economic justice and their working definition of exploitation,”^27^ which I, following others, expand to include other social groups.^28^ Scott’s analysis brought much-needed attention to the unquantifiable factors that prompt everyday forms of resistance and struggle. His work deftly illuminates how subjective conceptions of justice inform where social classes stand in the continuum between compliance and revolt. Nevertheless, his conceptualization of the moral economy has several important shortcomings, which still color uses of the term today. First, Scott situates the moral economy and the actions it engenders outside of and necessarily opposed to the market sphere, which it inevitably works to resist. This approach obscures the moral underpinnings of market-disciplinary projects, such as neoliberalism or liberal capitalism, and constructs a false dichotomy between noncapitalist and capitalist values and communities. It also homogenizes the moral economy, making the peasantry’s definitions of exploitation and justice appear both singular and uniform. Crucially for the purposes of this article, Scott’s *Moral Economy* and his subsequent *Domination and the Arts of Resistance* link oppositional practices to class position and agricultural resources.^29^ This connection fixes the forms of resistance that moral economies can engender in predetermined subversive scripts. It situates dissent amongst subordinate classes in a static theater of resistance, “social spaces of relative autonomy” or “offstage social spaces” somehow unaffected by hegemonic forces.^30^ In doing so, Scott neglects “the *realpolitik* of place and practice” through which social actors maneuver and fails to consider that the discursive and material boundaries in which they operate are themselves “effects of power.”^31^

During my time in Jordan (2013–15), bread subsidy preferences were justified by recourse to an array of values distilled through moral rubrics. In order to do their plurality and heterogeneity justice, this article situates moral economies of one subsistence good—bread—in multiple, crisscrossing fields of power.^32^ Anchored by a Gramscian understanding of hegemony, where political domination is always incomplete and subject to situated forms of struggle,^33^ this approach seeks to honor the normative complexity and historical development of human communities by eschewing rigid dichotomies between traditional and capitalist societies or economies.^34^ It will illustrate how popular grievances are not the product of overarching moralities or uniform economic imaginaries but lie instead at the level of specific, meaning-laden goods and practices.^35^ These grievances can engender resistance, but the latter is never articulated from a position or space untouched by power.^36^ Most importantly, this mode of analysis can better specify the mechanisms through which moral economies emerge and how they relate to popular unrest, hegemonic formations, and policy outcomes, not as separate fields but through their mutual imbrication.

## MOLDING EXPECTATIONS: THE HISTORY OF JORDAN’s BREAD SUBSIDY

Consumers, producers, and distributors of subsidized bread in Jordan are not passive recipients of singular cultural logics that determine their moral economies. Rather, they are highly reflexive when discussing bread and subsidy policy. Yet, as Ben Fine points out, individuals “are not reflexive in circumstances chosen by themselves.”^37^ Therefore, I treat the provisioning process of Arabic bread—production, distribution and consumption—as connected not only through cultural understandings of justice and appropriate economic practice, but also through material networks, historical struggles, institutional legacies, and political relationships.^38^ John Bohstedt conceptualizes these factors as comprising the “politics of provisions,” the crucial parameters that shape conflicts over subsistence goods.^39^ The structural factors encapsulated by Bohstedt’s “politics of provisions” are far from determinative, as the assertion and achievement of entitlements to public goods is an iterative process. Nevertheless, they do point to a catalogue of the expectable, the possible, and the desirable.^40^ As Scott argues, “Beyond . . . brute physiological needs, there is clearly an historical dimension to subsistence levels in which minimum standards bear some relation to previous experience.”^41^

Beginning in the late 1960s, the Hashemite regime began to provide certain commodities to various sectors of society. These efforts were led by Prime Minister Wasfi al-Tal, who hoped to “rationalize the foundations of Jordan’s patrimonial state.”^42^ By minimizing informal patronage practices while channeling social welfare and investment through government channels, al-Tal sought to foster broad-based development and nurture loyalty to the Hashemite monarchy at a time of rapid population growth and regional instability. Despite their initial promise, the results were mostly piecemeal, yet al-Tal’s initiatives did set a precedent for the state’s growing involvement in markets for strategic commodities.

Following the events of Black September (1970–71), the parochial nationalism on which the monarchy increasingly relied to legitimate its rule had to be palpably acted out.^43^ The impact of rampant inflation on the citizenry’s livelihood, coupled with the Hashemite regime’s desire for their orderly allegiance, helped institutionalize a commitment to supporting the purchasing power of consumers. These programs specifically targeted East Bank Jordanians on fixed salaries in the military and bureaucracy, which now formed the monarchy’s key base of support.^44^ In 1974, the Ministry of Supply was established and given responsibility for administering subsidies for politically sensitive goods.^45^ The government began to import a host of commodities, distributing them through a network of civil and military cooperatives in which retail prices were set.^46^ Subsidized products first included products such as wheat and sugar, and subsequently encompassed items such as tea, powdered milk, rice, and frozen poultry. Although initially intended only for members of the bureaucracy and Armed Forces, “informal reciprocal mechanisms” ensured that they benefited the populace as a whole.^47^ Eventually, most of these measures became universal consumer supports. Over the following fifteen years, the citizenry’s experience of receiving a range of commodities at accessible prices generated upward movement in subsistence expectations. This is the soil from which many Jordanians continue to judge the Hashemite regime’s provisionary practices.

Most food subsidies and price controls were minimized or eliminated in February 1992. These measures followed the resumption of a structural adjustment program first agreed to by the Jordanian government and the International Monetary Fund in 1989. Its initial implementation triggered riots in the southern town of Maʿan, which quickly spread throughout the country. Fearful of further bouts of unrest, the government provisionally lifted the wheat subsidy only in 1996. The change, meant to satisfy IMF loan requirements, set off bread riots in the city of Karak in August 1996. The price of bread reached twenty-five *qirsh* ($0.33) per kilogram before a new prime minister backtracked on the reform only months later.^48^ The subsidized price eventually settled at sixteen *qirsh* ($0.23) per kilogram of standard *khubz* ʿ*arabī*. Nevertheless, the wheat subsidy has not been impervious to international price fluctuations and broader processes of neoliberalization. The Hashemite regime faced one stern test following the dramatic rise in global commodity prices in 2007 when policymakers decided to stop subsidizing all wheat-based products. In early 2008, the subsidy system was altered so that only all-purpose unified flour of 78 percent extraction, known locally as *al-muwwahad* (unified flour), would be subsidized. New legislation made it illegal to use subsidized flour for anything but the cheapest *khubz* ʿ*arabī*. The government in turn largely avoided social unrest of the kind seen during previous changes by emphasizing the regressive nature of the universal wheat subsidy, which disproportionately supported the consumption of what are considered luxury goods: cakes, Arabic sweets (*baqla¯ wa, harīsa*), and more expensive varieties of bread (baguette, *khubz tanu¯ r, and khubz s_._a¯ j*).^49^ Economic analyst Husam Ayish described the cabinet’s strategy at the time as incredibly “smart,” as it justified reform through recourse to values of fairness, social justice, and equality.^50^

Since the 2008 restructuring, the government purchases wheat on the international market and sells it to private millers. The latter oversee the wheat’s conversion into different types of flour. The *al-muwahhad* variety, intended only for the production of standard *khubz* ʿ*arabī,* is then sold to government-approved bakeries at approximately one-seventh of its market price.^51^ Depending on their size and location, bakeries are allocated subsidized flour quotas by the Ministry of Industry, Trade, and Supply, which reimburses millers for the amount apportioned to bakers. *Khubz* ʿ*arabī* is then sold at the regulated price of sixteen *qirsh* ($0.23) per kilogram to any and all consumers. Notwithstanding its popularity, foreign donor preferences and fiscal pressures have combined to put the welfare program under increased scrutiny. Policy discussions following Jordan’s agreement to a thirty-six-month, $2.06 billion IMF loan in August 2012 included proposed changes to the universal bread subsidy.^52^ After liberalizing fuel prices in November 2012, the government began promoting a plan that would use electronic smart cards to counteract changes to the current policy.^53^ Only Jordanian citizens with valid identity cards would be given a monthly allowance to compensate for the difference between subsidized (JD 0.16 per kilogram) and predicted free market prices (JD 0.38 per kilogram). Bakery owners, consumer protection groups, political parties, and activist networks immediately remonstrated.^54^ Although the outcome of the subsequent debate—delay and nonreform—was foreseeable, views expressed during its progression provide an interesting window into Jordanian politics.

## BREAD AND BUEAU CRATS: MORAL ECONOMIES OF DISTRIBUTION

Jordan’s inance Ministry, a drab modernist office block, appears more like the beating heart of an interventionist bureaucracy born amid the ascendancy of state-led development than the sleek hub of business-friendly free marketeers. Yet during the last fifteen years, this ministry has been at the center of Jordan’s socio-economic transformation, the outpost of neoliberal crusaders bent on implementing a comprehensive set of pro-market reforms. Finance Minister Umayya Toukan received me in his office on the second day of the holy month of Ramadan. It was 30 June 2014, and the bread subsidy topic was once again in the news. A recent visit by IMF President Christine Lagarde had made headlines due to a brief verbal skirmish with Jordan’s Prime Minister Abdullah Ensour, who criticized the IMF for failing to consider local realities when pushing cutbacks in politically sensitive government expenditures.^55^ Toukan began by offering a brief history of consumer subsidies in the region, noting that during the hey-days of Arab Socialism governments throughout the Middle East had strategically chosen to support specific goods rather than target assistance to the poor. Problems emerged for non-oil-producing countries whenever global prices in commodities spiked, as governments were put under fiscal duress due to inflexible subsidy systems.

When asked about the current policy, the finance minister argued that universal subsidies inherently foster perverse incentives: “Currently, flour is wasted, smuggled, and used illegally. Government interventions inevitably create market distortions.”^56^ While most interviewees defended the bread subsidy precisely because it defied market logics, the former head of Jordan’s Central Bank described price controls as the source of “wasteful and inefficient outcomes.” Interestingly, Toukan’s stated preference for the free market was not solely based on macroeconomic considerations: “Inequality in outcomes are quite clear. The rich and highly profitable businesses benefit far more from subsidized goods than the poor, who do not get their fair share.” The finance minister’s neoliberal predilections are hardly amoral; he sees no gap between moral questions of justice and cold-blooded calculations of allocative efficiency.^57^ Benefits from free market reforms will eventually trickle down to all Jordanians, Toukan reasons, who are only harmed by governmental interventions that compromise efficiency, maximization of utility, and economic freedom.^58^

Despite the finance minister’s normative and macroeconomic inclinations, his remarks concerning bread were cautious: “The bread subsidy is an issue where politics and economics may not align with each other. Good policy may not be politically possible or desirable.” Finance minister Toukan represents the neoliberal wing of the Hashemite regime, a loosely tied amalgamation of technocrats, often foreign-educated and with personal ties to the king, who have dominated the country’s main economic decision-making bodies over the last fifteen years.^59^ Their moral economy is infused with neoliberal tropes that emphasize market efficiency, freedom to consume, and self-care as the path to economic liberty, collective productivity, and social justice. Nevertheless, various interviewees in this camp emphasized that economic reforms should stop short of endangering stability. Market-friendly alternatives proposed during the subsidy debate were considered problematic not due to the neoliberal logics they furthered but because their implementation could affect the political status quo. Toukan expressed this sentiment succinctly towards the end of the interview: “The at most 300 million dinars spent on the bread subsidy is not so much, not as much as the symbolism bread gives to the life of the people.” The normative raw material at the finance minister’s disposal can justify capitalist logics of exchange for a host of products and relations, just not *khubz* ʿ*arabī.*

The Ministry of Industry, Trade, and Supply (MOITS), which is responsible for the timely delivery of subsidized flour and the regulation of bakeries, took the lead in promoting the smart card proposal. When advocating this alternative, MOITS officials emphasized the disjunctures in a universal subsidy system that supports all consumers while fostering fraud amongst bakers and flour millers. Although there are no reliable estimates of leakages, government officials discuss flour wastage constantly.^60^ Yet in stark contrast to the finance minister, MOITS officials valued the government’s role in regulating markets; they simply sought to improve it. The limits placed on the complete marketization of bread were deemed judicious and necessary. For Samer Khouri, director of market control for MOITS, “the ministry preserves social integrity through the subsidy since bread is the food of the poor.”^61^ The welfare program should be reformed, he and other ministry officials affirmed, but not to reflect market outcomes. Instead, changes should benefit poor citizens, who are the most reliant on government interventions. Engineer Emad al-Tarawneh, director of rrade and strategy at MOITS, argued that despite the current system’s many faults, some form of consumer support is necessary: “It is our obligation to help citizens subsist; we do so through bread because it is part of our culture.”^62^ Essa Al-Dmour, MOITS Director of Internal Audits, who holds primary responsibility for spending related to the bread subsidy, similarly emphasized that, “The subsidy mechanism must undoubtedly be changed. It disproportionately helps wealthy bakery and restaurant owners. But support for consumers must continue, it is our responsibility to the lives and well-being of the citizenry [*rafa¯h wa h_._aya¯t al- ^muwa¯^.^tinīn^*].”^63^ For MOITS officials, the bread subsidy demonstrates the munificence of the Hashemite regime, a necessary corrective to market outcomes and a key pillar of “social justice and governmental responsibility held sacred by all Jordanians.”^64^ They draw mainly on values of compassion and solidarity, expressed through a civic register, to defend the citizenry’s collective entitlement to one subsistence good through which life is made livable.

This moral economy, which implicitly links social justice and regime legitimacy to the availability of subsidized bread, is also prominent among several political and community groups. MP Amjad Majali, head of the centrist parliamentary bloc al-Nahda (the Renaissance), was one of the more vociferous opponents of subsidy reform since it was first proposed. On 21 May 2015, he expressed publicly sentiments he had relayed to me in a personal interview, asserting that, “We must remind the prime minister that bread is not caviar nor salmon.”^65^ Majali went on to emphasize the product’s importance to Jordan’s poor and working classes, whose survival would be threatened without access to the foodstuff.^66^ Similarly, the Federation of Independent Trade Unions consistently opposed subsidy reform because of how it could affect Jordanian living standards.^67^

Analogous remarks could be made about a host of other promarket policies recently enacted by the government. Yet, the meanings with which bread has been imbued make it the ideal marker through which actors who favor modest levels of redistribution and oppose neoliberal measures can mobilize popular opinion and express legitimate forms of dissent. Although most of these actors support the monarchical system as currently constituted, the values on which they draw to make their claims are far different than those that increasingly dominate official institutions.

Then-Senator Hani Mulqi best exemplifies the prevalent position amongst members of the security forces, high-level government officials, and prominent politicians closely allied with the palace.^68^ In an interview, Senator Mulqi emphasized tacit support for most IMF prescriptions, yet argued for leniency in this case. When pushed on the potential pitfalls of subsidy reform, Mulqi stated:

This is bread! The current situation, both regional and domestic, means that we cannot play with the bread subsidy, notwithstanding the inefficiencies that permeate the current system. The government correctly fears the political price it will pay if reform is attempted. Bread is the food of the poor; it symbolizes life and ensures social stability. This cultural significance makes reform dangerous.^69^

Mulqi’s position broadly reflects that of the palace’s closest political allies, more interested in monarchical survival than intellectual coherence. The ideological permutations this engenders have elsewhere been described as “conservative royalism,”^70^ “fuzzy nationalism,”^71^ “Hashemitism,”^72^ or a “peculiar version of modern dynasticism.”^73^ Among its proponents, social stability is portrayed as a supreme good, both economically beneficial and politically necessary.^74^ As King Abdullah II asserts, “Without security and stability and the rule of law there can be no development, no modernization, and no progress.”^75^

Fearful of social unrest and regional instability, the Palace promotes this “Hashemitist” political rationality so as to realign conceptions of citizenship to ensure its hegemony.^76^ It emphasizes social stability, national unity, and benevolent monarchism as the underpinnings of Jordanian collective identity and politics.^77^ Yet crucially, publicity campaigns emphasize not just the need for national unity and allegiance to the monarchy but also the regime’s commitment to social harmony, individual dignity, and the fight against poverty, all as part of a broader effort to nurture political loyalty among Jordanians.^78^ King Abdullah II’s Speech to the Second Ordinary Session of the seventeenth parliament in November 2014 illustrates such logics. After mentioning regional turmoil and threats to national security but before turning to “several mega projects” and the need for expedited legislation pertaining to “energy and investment,” the king emphasized how the country’s economic blueprint “aims at achieving decent living standards and a promising future for the sons and daughters of our beloved Jordan.”^79^ As Mulqi put it in far starker terms, “Many people depend on the state to eat, for their survival. The king knows this.”^80^ Despite their tactical ambiguity, such pronouncements position the king as the guarantor of subsistence, the patron of loyal citizens who are in need. The monarchy’s implicit emphasis on reciprocity and obligation between rulers and ruled means it cannot allow for social relations to be mediated exclusively by the market. Hence, the economic protections expected by Jordan’s lower classes are precisely those responsibilities that the palace appears to accept. Indeed, various government sources of this persuasion indicated that the king himself had intervened to put an end to the subsidy reform debate. News reports consistently emphasized that, like these officials, the monarch also considered bread “a red line.”^81^ Similar to other instances, exceptions to the neoliberal trend were depicted as a “gift from above,” the father of the kingdom taking care of “his people.”^82^

All the government officials I queried recognized bread’s multifaceted importance. Nevertheless, the normative commitments that shaped their distributive preferences were far from similar. Policy inclinations were justified by recourse to an array of values, distilled and interpreted through moral rubrics. Yet crucially, discussions of the bread subsidy were almost always colored by tropes present in the regime’s legitimating discourse. The fuzziness and internal diversity of this political rationality, which only the monarchy can fully encapsulate,^83^ helps explain why moral economies of bread among government officials are highly disparate; actors can choose to stress different values to rationalize or express their policy preferences while emphasizing their allegiance to the monarchical order.^84^ The palace’s intermittent use of neoliberal, tribal, populist, and Islamist vocabularies, which cohere around monarchy, national unity, and social stability, aptly illustrates what Foucault describes as the “tactical polyvalence of discourse,” which is best analyzed as “a series of discontinuous segments whose tactical function is neither uniform nor stable . . . a multiplicity of discursive elements that come into play in various strategies.”^85^ Although it aids the palace’s hegemony through its calculated inclusivity, such polyvalence also allows certain discursive elements to be reappropriated and resignified by the very citizens they seek to interpellate.

## OUR DAILY BREAD: CAUTIOUS RESISTANCE AND CALCULATED CONFORMITY

Saʿid, the resident of Jabal al-Nadhif mentioned in the introduction, lies at the mercy of nebulous market forces. Without a high school diploma, opportunities in the formal sector are unavailable. As a Jordanian citizen, he is unattractive to the small businesses that prefer employing cheaper migrant labor. Saʿid has access to family networks and neighborhood organizations that ensure he will not starve. Yet ultimately, his primary source of daily calories and life-sustaining food is the combination of ministry officials, flour millers, and bakers who ensure subsidized bread’s availability. The foodstuff marks his daily rhythms and is crucial to his subsistence. It is a primary ingredient in his snacks of *khubz wa-shay* (bread dipped in tea), a crucial element in the JD 0.30 falafel sandwich he buys for lunch and his main contribution to family meals. When describing the bread subsidy, Saʿid drew upon vocabularies and values frequently used by ordinary citizens but also deployed by those in power. “In the *hadīth*, *khubz* embodies a responsibility of those in power. Today, the bread subsidy is how the regime demonstrates its commitment to fairness and compassion,”^86^ he reasons. Saʿid emphasized the foodstuff’s links to religious values he held dear such as *ihsa¯ n* (beneficence) and *s_._adaqa* (almsgiving). He negotiates these values through moral rubrics, which work to produce a moral economy in which bread’s accessibility was deemed an obligation of state authorities.

Saʿid’s remarks were quite different from Ahmad’s, a lower middle-class professor at one of Amman’s burgeoning Arabic-language institutes. By ensuring the timely distribution of flour, Ahmad argues, the government combats the dissolution of the social bonds that give Islamic societies both meaning and order: “*al-* ʿ*ayīsh* (lit. “life,” but by extension, “bread”) is a pillar of our everyday existence; it is part of our culture and religion.” He prizes bread not solely because of its caloric content but also due to its links to systems of meaning he holds dear. In this regard, Ahmad asserts that the subsidy program allows for business practices that more closely correspond to Islamic conceptions of property and trade. “The bakery is a beautiful place,” he commented, after we stopped to purchase some subsidized bread on a trip to Mafraq, “Profits and prices are never excessive. It is a beacon of stability that gives us [consumers] peace of mind.” Many bakers I interviewed echoed this view, especially in the poor neighborhoods of East Amman. While most, like Ahmad, avoided criticizing the Hashemite regime, they made clear that neoliberal reforms were dissolving communal bonds and augmenting inequalities.^87^ They frequently portrayed subsidized bread as an essential material (*ma¯da a¯sa¯siyya)* whose low price was vital not only to the citizenry’s survival but also to up-holding the regime’s moral or religious obligations. Islam here functions not just as a matter of personal conviction or individual ethics, but also as a set of guidelines for ordering economic life.^88^ Although King Abdullah II does not appeal to Islamic precepts as often as his father did, he does emphasize—as do the country’s textbooks—Islam as a key pillar of Hashemite legitimacy.^89^ The monarchy’s occasional recourse to a religious register is what makes Ahmad and Saʿid’s remarks acceptable, potent, and subversive, all at the same time.

For Hani, a student at the University of Jordan and a member of the country’s Communist Party, bread is a nutritional staple whose subsidy is a right achieved through social struggle: “The policy is hardly an expression of benevolence, [as] the government knows it will face a backlash if it eliminated the subsidy.” Interestingly though, his critique forgoes revolutionary demands and draws on established principles to make its claims. For example, Hani stressed the regime’s emphasis on communal well being and social justice when appraising potential subsidy reform.^90^

Bread is the fundamental element of every poor person’s diet, which is why it is so important. Without it, we could not survive and would be left completely at the mercy of capitalism. There can be no justice, no security, stability or survival if the people cannot eat. Bread makes life possible. This is why we defend it. The regime itself tells us it is a red line.

Umm Hassan, a high school teacher and self-identified socialist similarly emphasizes “national values” to decry reform efforts. Her critique is clothed in hegemonic dress: “Without bread we could not make do. Jordan would no longer be the country of safety and security [*balad al-a¯ min wa-l-a¯ yma¯ n*] those in power love to acclaim.” Subsidized bread holds pride of place in Umm Hassan’s notion of economic justice and definition of exploitation. Such views are indicative of the stake that residents of the country continue to have in retaining distributive provisions controlled, organized, and financed by state authorities. Accessing bread is not only about acquiring a particular foodstuff, but also about sustaining community ties, striving for equitable distribution of public goods, and demanding recognition from those in power. Both the Hashemite regime and citizenry’s usage of *khubz* as a metaphor for subsistence, stability, and compassion have reinforced these communal understandings of the foodstuff in the Jordanian socio-cultural context.

Among consumers, support for the bread subsidy is justified in terms of values including solidarity, justice, and beneficence. These values, and the diverse moral economies they undergird, are articulated through religious, civic, and political registers, sometimes in combination. Despite their diversity, moral economies among poor and middle-class citizens all come together to imbue bread with meanings that make subsidy a locus for popular grievances. The latter were not articulated “offstage” or through traditional, noncapitalist precepts. Rather, the links fashioned between bread and the Hashemite regime’s own rhetoric, given how malleable its terms are, offered useful tools through which to ground opposition to subsidy reform.^91^ Ordinary citizens could mitigate the risks of confrontation and thus declare their opinions openly.^92^ Despite undeniable asymmetries in power, the polyvalence of the monarchy’s legitimating discourse furnished ordinary citizens with a potent vocabulary with which to criticize policy proposals that threatened their right to subsistence. They were ever-vigilant wardens, rather than prisoners, of the Hashemite regime’s rhetoric. Reducing interactions between ordinary citizens and elites to a binary of exploiters versus resisters oversimplifies the multiple and shifting interfaces that characterize such exchanges.^93^

## BAKERS: PRODUCTION AND MORALITY IN THE PURSUIT OF PROFIT

Cognizant of their centrality to daily subsistence, bakery owners who ran operations vastly different in terms of size, scope, and success described bread and justified their policy preferences through an array of moral commitments. In Jabal al-Nadhif, Hamdi Barjus argued that the *wa*ẓ*ifa ijtima¯* ʿ*iyya* (social function) of private property delineated in the hadith is meant to ensure that the pursuit of profit does not harm social harmony by keeping acquisitive instincts in check: “I sell only subsidized bread, always at the same price and without corruption, to demonstrate my lack of interest in excessive, unnecessary profits.”^94^ Barjus credits the government with combatting exploitation and injustice through the timely distribution of subsidized flour, a position he seeks to affirm through his own business practices. Al-Sharq offers an intriguing contrast. A large-scale operation in the wealthy West Amman neighborhood of Dabuq, the bakery sells a dizzying array of pastries, cakes, and fancy breads, from which most of the retailer’s profits derive. Its owner, engineer Rachid al-Homsi, also portrays bread and its subsidy through a religious register: “Bread is sacred. The subsidy is the government’s version of *zaka¯ t*, it ensures that the poor can survive but it does not preclude honest profits made from other products.” Both Barjus and al-Homsi articulate their moral economies in a religious register. Yet the impact of Islamic values upon their business practices is very different. The latter owe as much to shifting temporal manifestations of geography and social class as they do to any values defined as uniquely Islamic.^95^ Contrary to the perception that Islamic vocabularies and attachments replace situated ethics, questions of morality are problematized and worked through in particular situations and milieus as mediated by values. Nonetheless, such differences did not preclude support of some form of bread subsidy, which they and the majority of bakers saw as pivotal to communal dignity and individual survival.

The Bakery Owners Association (BOA) offered the most intriguing layering of values when discussing bread. Charged with representing this incredibly diverse constituency, the BOA emphasized social solidarity and cohesion, governmental responsibility, and sensitivity to endemic poverty in the media and other public forums.^96^ These tropes were deployed alongside the kind of promarket rhetoric usually associated with neoliberal discourses. For example, BOA President Abdul Illah Hamawi argued that, by “providing the strategic good of bread to the citizenry,” bakers help ensure social peace and stability, values stressed by conservative monachists.^97^ This layering is evident in the organization’s policy recommendation. To improve the subsidy’s “efficiency,” the BOA proposes a neoliberal technique—a three-year transition to a free market in production.^98^ This strategy would minimize government intervention in bakers’ business practices. At the same time, the organization favors a remunerative measure not strictly driven by market logics: direct cash transfers for citizens so as to protect their purchasing power.^99^ Throughout the debate, the BOA underscored its “commitment to bearing its national and moral responsibilities,” while promoting what it described as the “best option for people as it preserves their dignity and achieves significant savings for the state treasury.”^100^ Although the values it stressed and vocabularies it deployed depended on the audience with which it was engaging, the BOA never failed to frame its position in ways that could cajole both the citizenry and policymakers.

Values circulated through Sunni Islam, Arab socialism, neoliberalism, and conservative royalism influence the way distributors, consumers, and producers portray the provisioning of bread in Jordan. These discourses establish the parameters and boundaries within which individual and collective actors express themselves. Importantly, however, the values they advocate are negotiated through moral rubrics that allow for interpretive flexibility. This flexibility engenders a highly diverse set of moral economies, the vast majority of which ascribe to bread politically salient meanings. These meanings are crucial to understanding why subsidy reform proposals are seen by citizens as threats to not only individual survival but also to social stability and a perceived right to subsistence. They also help explain why resistance to subsidy reform takes the shape it does. Unlike discussions of King Abdullah’s right to rule or the role of the secret police in politics, opponents of subsidy reform did not opt for disguised dissent or Scott’s “weapons of the weak.” Rather, their critiques sought to attract attention and sway advocates amongst the powerful. These forms of “rightful resistance” can hamstring those in power precisely because they are set within the confines of established values and rhetoric of the ruling order, making them very hard to disavow.^101^ Crucially however, the story of rightful resistance to subsidy reform is not simply one of a struggle for the symbolic high ground. If perceptions of injustice, value-laden arguments, and shared moral economies were the sole determinants of Jordanian social policy, the latter would look very different. The persistence of the bread subsidy also stems from the material networks, social relationships, and historical struggles that comprise the politics of provisions.

## SUBSIDIZED BREAD AND THE POLITICS OF PROVISIONS

On various occasions in Jordan’s recent history, most notably the country-wide unrest in April 1989 and the Karak bread riots of 1996, political parties, activist groups, and neighborhood networks mobilized to oppose cuts to long-standing welfare programs and consumer supports.^102^ The accelerated implementation of promarket measures under King Abdullah II (1999–) has not eradicated this vociferous opposition.^103^ Increased unemployment and downward pressure on wages engendered by neoliberalization have only increased poor and working-class vulnerability to shifts in the price of subsistence goods. As Asef Bayat argues, this process has transformed popular demands throughout the Middle East.^104^ Past struggles over wages and working conditions have shifted to issues of collective consumption—housing, electricity, water, and food.

Most recently, the Jordanian Hirak (movement) of 2011–13 gave voice to rising discontent in the small towns and villages of the East Bank, long (mis)considered bastions of monarchical support. Combining disgruntled military veterans, government employees, and loose networks of young activists, the movement coalesced around opposition to corruption, cuts in public employment, and subsidy reform.^105^ The issues around which the Hirak rallied are hardly happenstance. Ideological divisions, disputes over national belonging, and the complexity of Jordan’s class structure militate against collective action on most issues. The regime’s institutional strategies coupled with its links to tribal and economic elites fostered through kinship and patronage make the formation of any broad-based opposition coalition difficult.^106^ Yet, the meanings with which bread has been imbued and the moral economies that undergird its significance make it a powerful symbol through which Jordanians can and have historically transcended the divisions that consistently hamper wide-ranging resistance to economic reforms. Whereas unified opposition to the country’s electoral law, privatization of public spaces, or restriction of labor rights requires elaborate organization and framing efforts, most of which have failed, shared resolve regarding subsidies for crucial subsistence goods has been far easier to achieve. From Islamic centrists to Marxists, conservative nationalists to Muslim Brothers, not one political organization of note publicly supported bread subsidy reform during the most recent debate. Undoubtedly, their reticence was linked to grievances engendered by moral economies in which *khubz* ʿ*arabī*’s availability was linked to a perceived right to subsistence and portrayed as an obligation of state authorities. These certainly leavened popular apprehensions, drove societal angst, and lubricated its expression. Yet the effectiveness of opposition to bread subsidy reform also lay with the horizontal networks that enabled citizens to act collectively and exert leverage, as well as the vertical ones that allow their claims to be made, and to succeed.^107^

Jordan’s authoritarian incumbents are hardly immune to such pressures. Public unrest and vociferous opposition in the face of austerity measures perceived to be unjust have long forced the monarchy to navigate various power centers throughout the body politic. The palace and its allies know full well that violating what amounts to an implicit pact of subsistence entails not only moral denunciations, but also the distinct possibility that popular grievances may lead to social unrest.^108^ To this day, they make sure that the state’s institutional apparatus fulfills certain obligations. Foremost among these are social expenditures that help the citizenry subsist. By upholding the bread subsidy, elites are seen to respond to the normative and physical claims of the community. They do so through a concrete policy whose daily manifestation and importance is inescapable, fulfilling a crucial obligation to the lower classes whose compliance they elicit.^109^ For the Hashemite regime, it remains cheaper and easier to appease marginalized citizens than to crush them, both materially and morally. In spite of an undoubted commitment to an extreme set of promarket economic policies, monarchical legitimacy still depends somewhat on a distorted but enduring system of social provision, which access to grants, loans, and loan guarantees from external backers help make possible.

At the same time, the bread subsidy debate helps illuminate the nature of opposition politics in Jordan. In brief, those uneasy or critical of promarket measures can articulate their disapproval openly while largely remaining embedded within the monarchy’s chosen frames of reference. Even as they contest reform, rightful resisters tie the availability of subsidized bread to values emphasized in the regime’s legitimating rhetoric, whose “fuzziness” and discursive polyvalence means it can accommodate but also must respond to certain popular grievances.^110^ This dynamic perpetuates a central myth of Jordanian politics. Protestors and opposition elements act as if the government enacts unpopular economic measures, rather than the king. This separation elucidates why the vast majority of public protests can criticize the government, avoid the implementation of certain policies, and even bring down a prime minister, but not question the role of the monarchy.^111^ Of course, delaying the full commodification of bread is itself an important accomplishment. Yet, by clothing themselves in hegemonic dress, most opponents of subsidy reform leave the broader features of market reform and authoritarian power intact. This outcome does not require the citizenry’s uncontested acceptance of the monarchy or its vocabularies of choice, but rather the repackaging of resentments, critiques, and resistance in a fashion that may very well contribute to the Hashemite monarchy’s legitimacy and project of rule.^112^

## CONCLUSIONS

After years of neglect, scholars have recently turned their attention to issues of food politics in the Middle East and North Africa. Despite this empirical shift, the predominantly policy-centric lens through which food has been analyzed suffers inevitable limitations. Most contemporary works scrutinize causal links between increases in world food prices and the onset of popular protests during the heady days of the so-called “Arab Spring.” Such approaches frequently place causal priority on regime type, class dynamics, or commodity prices, bracketing off culture, discourse, and other unquantifiable factors from their frameworks. These perspectives are incomplete. They fail to acknowledge that “systems of social provision are embedded in moral and symbolic orders that shape their development.”^113^

In contrast, this paper has focused on the microprocesses that imbue one foodstuff with meaning. It elucidates the process through which certain values, negotiated by way of flexible rubrics, shape moral economies where the provision of subsidized bread is linked to a perceived right to subsistence. I have done so not to reify a homogeneous spatio-temporal community enraptured by religion or traditional modes of exchange, but to demonstrate how the subjectively meaningful properties and moral economies of *khubz* ʿ*arabī* are embedded in ever-shifting social relations, “not in generalized mechanical moralities or romanticized pasts.”^114^ Although briefly, I have tried to place these moral economies in relation to the “politics of provisions” so as to understand subsidy reform’s links to neoliberalization, opposition politics, and authoritarian power. By taking both moral economies and the politics of provisions into account, scholars can better account for the pivotal factors that give moral axioms related to subsistence goods or welfare programs both traction and effectiveness.^115^ We can better understand why particular grievances emerge and how they engender certain types of social mobilization and criticism. When anchored in a Gramscian understanding of hegemony, in which social actors maneuver within cross-cutting fields of force, these two concepts become less a framework valid for all times and places and more a set of tools through which to analyze policy debates and deliberations during particular historical junctures.^116^ In Jordan, this approach clarifies not only why popular grievances coalesce around bread, but also how opposition to subsidy reform relates to the institutional fabric and discursive formations in which it is embedded. It is in this crucial terrain, not in the overt collective defiance of power holders or in the complete compliance of the citizenry, where the limits of Jordan’s existing political relationships are consistently probed, both from “above” and “below.” Over the last twenty-five years, this battle has consistently favored the country’s tribal elites, well-connected capitalists, and authoritarian incumbents, though not without important exceptions. Hegemony can be thin or thick, but the fissures, tensions, and contradictions that inhabit all political orders ensure it is contested, and never complete.

## NOTES

1 Anne Marie Baylouny, “Militarizing Welfare: Neo-Liberalism and Jordanian Policy,” The Middle East Journal 62 (2008): 277–303

2 Christopher Parker, “Tunnel-bypasses and Minarets of Capitalism: Amman as Neoliberal Assemblage,” Political Geography 28 (2009): 118.

3 Jumanah Ghneimat, “Ayn Maqula ‘al-Khubz Khatt Ahmar’?!,” al-Ghad, 17 September 2013, accessed 10 December 2017,

4 George Barham, “al-Naʾib al-Majali: al-Khubz Laysa Kafiyaran wa-La Salmun,” Roya News, 21 May 2015, accessed 7 December 2017, .

5 Interview with the author, 9 July 2014, Amman.

6 “Lan Tuzid al-Khubz fi Lisan Wahidan ʿala al-Muwatin,” al-Dustur, 17 June 2015, 8; “Lan Tarfʿa Saʿar al-Khubz ʿala al-Muwatin Itlaqan,” al-Dustur, 28 November 2014, 12.

7 Sean L. Yom, “The New Landscape of Jordanian Politics: Social Opposition, Fiscal Crisis, and the Arab Spring,” British Journal of Middle Eastern Studies 42 (2015): 284–300.

8 Lara Deeb and Mona Harb, “Choosing Both Faith and Fun: Youth Negotiations of Moral Norms in South Beirut,” Ethnos 78 (2013): 4; Tania Murray Li, The Will to Improve: Governmentality, Development, and the Practice of Politics (Durham, N.C.: Duke University Press, 2007), 22.

9 Aihwa Ong, Neoliberalism as Exception: Mutations in Citizenship and Sovereignty (Durham, N.C.: Duke University Press, 2006), 199.

10 ʿAla al-Qiraleh, “al-Hamawi: Iritfaʿa Istihlak al-Khubz 15% Muqarana maʿa al- ʿAm al-Madi,” al-Raʿi, 21 August 2014, accessed 6 December 2017, http://www.alrai.com/article/665039.html.

11 World Food Programme, “Jordan Food Security Survey in the Poverty Pockets, August-September 2008,” accessed 12 May 2014, http://documents.wfp.org/stellent/groups/public/documents/ena/wfp204530. pdf.

12 José Ciro Martínez, “Bread is Life: The Intersection of Welfare and Emergency Aid in Jordan,” Middle East Report 272 (2014): 30–35.

13 Benjamin Orlove and Ella Schmidt, “Swallowing Their Pride: Indigenous and Industrial Beer in Peru and Bolivia,” Theory and Society 24 (1995): 271–98. One well-known example is the baguette, which became a crucial conveyor of social identity and “metonym for the nation and its civilization in France.” Steven Laurence Kaplan, The Bakers of Paris and the Bread Question, 1700–1775 (Durham, N.C.: Duke University Press, 1996), 3.

14 Emily Ohnuki-Tierney, Rice as Self: Japanese Identities through Time (Princeton, N.J.: Princeton University Press, 1994), 9.

15 Jane K. Cowan, Dance and the Body Politic in Northern Greece (Princeton, N.J.: Princeton University Press, 1990), 132.

16 Erica Simmons, “Grievances Do Matter in Mobilization,” Theory and Society 43 (2014): 532.

17 For an interesting analysis of rice’s role in fostering collective identity in Japan, see Emiko Ohnuki-Tierney, “Structure, Event and Historical Metaphor: Rice and Identities in Japanese History,” Journal of the Royal Anthropological Institute 1 (1995): 227–53. In Mexico, corn tortillas have also been framed as being emblematic of the people, often in ways that resonate powerfully throughout the country. For more see, Erica Simmons, “Corn, Markets, and Mobilization in Mexico,” Comparative Politics 48 (2016): 413–31.

18 James C. Scott, Seeing Like a State: How Certain Schemes to Improve the Human Condition Have Failed (New Haven, Conn.: Yale University Press, 1998), 1–2; Marisa Wilson, Everyday Moral Economies: Food, Politics and Scale in Cuba (London: John Wiley & Sons, 2013), 79.

19 Jürgen Habermas, The Lure of Technocracy (London: John Wiley & Sons, 2015), 21.

20 Michael Lambek, “The Anthropology of Religion and the Quarrel between Poetry and Philosophy,” Current Anthropology 41 (2000): 315.

21 James C. Scott, Weapons of the Weak: Everyday Forms of Peasant Resistance (New Haven, Conn.: Yale University Press, 1985), 235.

22 Following Shielke, the term “registers” is used to convey the performative and highly situational character of norms. Samuli Schielke, “Ambivalent Commitments: Troubles of Morality, Religiosity and Aspiration among Young Egyptians,” Journal of Religion in Africa 39 (2009): 166.

23 Lara Deeb, and Mona Harb, Leisurely Islam: Negotiating Geography and Morality in Shi’ite South Beirut (Princeton, N.J.: Princeton University Press, 2013), 18.

24 John Bohstedt, “The Moral Economy and the Discipline of Historical Context,” Journal of Social History 26 (1992): 265–84; E. P. Thompson, Customs in Common (London: Merlin Press, 1991).

25 For more on how Scott’s use of moral economy differs from that of E.P. Thompson, see Marc Edelman, “Bringing the Moral Economy Back in. . . to the Study of 21st-Century Transnational Peasant Movements,” American Anthropologist 107 (2005): 331–45.

26 James C. Scott, The Moral Economy of the Peasant: Subsistence and Rebellion in Southeast Asia (New Haven, Conn.: Yale University Press, 1976), 35.

27 Ibid., 3.

28 Thomas Clay Arnold, “Rethinking Moral Economy,” American Political Science Review 95 (2001): 85– 95; William James Booth, “On the Idea of the Moral Economy,” American Political Science Review 88 (1994): 653–67; Andrea Muehlebach, The Moral Neoliberal: Welfare and Citizenship in Italy (Chicago: University of Chicago Press, 2012).

29 James C. Scott, Domination and the Arts of Resistance: Hidden Transcripts (New Haven, Conn.: Yale University Press, 1990).

30 Ibid., 118.

31 Donald Moore, “Subaltern Struggles and the Politics of Place: Remapping Resistance in Zimbabwe’s Eastern Highlands,” Cultural Anthropology 13 (1998): 351. Scott’s later work and his ground-level illustrations are more attuned to the dynamic context in which oppositional practices take place. For his own defence against the charge made above, see James C. Scott, “Afterword to ‘Moral Economies, State Spaces, and Categorical Violence,’” American Anthropologist 107 (2005): 395–402.

32 Benjamin S. Orlove, “Meat and Strength: The Moral Economy of a Chilean Food Riot,” Cultural Anthropology (1997): 234–68.

33 My reading of Gramsci here draws heavily on John Chalcraft, Popular Politics in the Making of the Modern Middle East (Cambridge: Cambridge University Press, 2016), 29–45.

34 Murray Li, The Will to Improve, 22–25; Peter Jackson, Neil Ward, and Polly Russell, “Moral Economies of Food and Geographies of Responsibility,” Transactions of the Institute of British Geographers 34 (2009): 12–24.

35 Arnold, “Rethinking Moral Economy,” 85–95; Simmons, “Grievances Do Matter,” 513–46.

36 Tania Murray Li, “Governmentality,” Anthropologica 49 (2007): 275–81.

37 Ben Fine, “Consumption Matters,” Ephemera 13 (2013): 226.

38 Wilson, Everyday Moral Economies, 21; Ben Fine, The World of Consumption: The Material and Cultural Revisited (London: Routledge, 2002); Jackson, Ward, and Russell, “Moral Economies,” 12–24.

39 John Bohstedt, The Politics of Provisions: Food Riots, Moral Economy, and Market Transition in England, c. 1550–1850 (Farnham: Ashgate Publishing, 2013).

40 Bohstedt, The Politics of Provisions, 7.

41 Scott, The Moral Economy, 17.

42 Paul Kingston, “Rationalizing Patrimonialism: Wasfi al-Tal and Economic Reform in Jordan, 1962–67,” in The Resilience of the Hashemite Rule: Politics and the State in Jordan, 1946–67, ed. Tariq Tell (Amman: Centre d’etudes et de recherche sur le Moyen-Orient Contemporain, 2001), 115– 44.

43 Joseph A. Massad, Colonial Effects: The Making of National Identity in Jordan (New York: Columbia University Press, 2001), 13–14; Russell Lucas, “Side Effects of Regime Building in Jordan: The State and the Nation,” Civil Wars 10 (2008): 281–93.

44 For more on this period, see Baylouny, “Militarizing Welfare,” 288–89; Massad, Colonial Effects, 204– 16, 240–57.

45 Lamis Andoni and Jillian Schwedler, “Bread Riots in Jordan,” Middle East Report 201 (1996): 40–42.

46 Baylouny, “Militarizing Welfare,” 291.

47 Ibid.

48 The prereform price was eight qirsh ($0.11).

49 Senator Jawad Anani, former minister of supply, minister of labor, deputy prime minister and foreign minister, interview with the author, 27 May 2014.

50 As quoted in Ala al-Furwati, “Darabka fi al-Makahbiz wa-Ghadab fi al-Shariʿa baʿd Rafʿa al-Daʿm ʿan al-Tahin,” al-Sijil 2, 15 November 2007, accessed 20 November 2015, http://www.al-sijill.com/sijill_items/ sitem86.htm.

51 During most of 2013, subsidized flour cost Jordan’s bakers thirty-five dinars per ton ($49), while the international market price hovered around 301.5 dinars ($426). These prices are set using a confidential formula which takes into account input costs (electricity, labor, rent, salt, and water) and ensures bakers a profit of 9 percent on every ton of flour made into khubz ʿarabī.

52 World Bank, Hashemite Kingdom of Jordan: Options for Immediate Fiscal Adjustment and Longer Term Consolidation (report by the World Bank, No. 71979-JO, Washington, D.C., 2012).

53 Omar Obeidat, “Government Mulls Cash Transfers via Smart Cards for Fuel, Bread Subsidies,” Jordan Times, 18 September 2013.

54 Tarek al-Daʿjeh, “al-Makhabiz: Tatbiq al-Bitaqa al-Dhakiya Yatasabab bi-Fawda wa-Yarbak al- Muwatinin,” al-Ghad, 16 September 2013, accessed 8 December 2017,

55 Samir Ghawi, “Public Support for Reform is Necessary—Ensour, Lagarde,” Jordan Times, 12 May 2014.

56 Ummaya Toukan, finance minister of the Hashemite Kingdom of Jordan, interview with the author, 30 June 2014, Amman.

57 For more on the normative underpinnings of neoliberalism, see Luc Boltanski and Laurent Thévenot, On Justification: Economies of Worth, trans. Catherine Porter (Princeton, N.J.: Princeton University Press, 2006).

58 There is a long tradition within liberalism that praises the moral benefits of society when it is run according to market principles. For more, see Marion Fourcade and Kieran Healy, “Moral Views of Market Society,” Annual Review of Sociology 33 (2007): 285–311.

59 For more on this this group, see Oliver Schlumberger and Andre Bank, “Succession, Legitimacy, and Regime Stability in Jordan,” The Arab Studies Journal (2001): 50–72; and Najib B. Hourani, “Urbanism and Neoliberal Order: The Development and Redevelopment of Amman,” Journal of Urban Affairs 36 (2014): 634–49.

60 Samer Khouri, director of market control of the Ministry of Industry Trade and Supply, interview with the author, 7 April, 2014, Amman; Hatem Halwani, minister of industry, trade, and supply of the Hashemite Kingdom of Jordan, interview with the author, 5 June 2014, Amman.

61 Halwani, interview with the author, 7 April 2014, Amman.

62 Halwani, interview with the author, 14 May 2014, Amman.

63 Halwani, interview with the author, 8 April 2014, Amman.

64 Ibid.

65 Barham, “al-Naʾib al-Majali.”

66 Ibid.

67 Ittihad al-Niqabat, al- ʿAmaliyya al-Mustaqilla, “Tajdir min Raf ʿa asar al-Khubz,” 14 May 2015, accessed 20 September 2015, https://www.assawsana.com/portal/pages.php?newsid=216096.

68 Mulqi is a former minister of trade and foreign affairs and was named head of the Aqaba Special Economic Zone Authority in November 2014. He currently serves as caretaker prime minister in the lead-up to the September 2016 elections.

69 Senator Hani Mulqi, interview with the author, 4 May 2014, Amman.

70 Curtis R. Ryan, “Political Opposition and Reform Coalitions in Jordan,” British Journal of Middle Eastern Studies 38 (2011): 367–90.

71 Hillel Frisch, “Fuzzy Nationalism: The Case of Jordan,” Nationalism and Ethnic Politics 8 (2002): 86–103.

72 Stefanie Nanes, “Hashemitism, Jordanian National Identity, and the Abu Odeh Episode,” The Arab Studies Journal 18 (2010): 162–95.

73 Andrew Shryock, “Dynastic Modernism and Its Contradictions: Testing the Limits of Pluralism, Tribalism, and King Hussein’s Example in Hashemite Jordan,” Arab Studies Quarterly (2000): 59.

74 Hasan Abu Nimah, “Jordan Is Strong, Safe and Stable,” Jordan Times, 25 February 2014.

75 His Majesty King of Jordan Abdullah II, “Remarks at the Celebration of Accession to the Throne, the Anniversary of the Great Arab Revolt and Army Day,” Amman, 8 June 2010, accessed 10 February 2015, http://kingabdullah.jo/index.php/en_US/speeches/view/id/435/videoDisplay/0.html.

76 José Ciro Martínez, “Leavening Neoliberalization’s Uneven Pathways: Bread, Governance and Political Rationalities in the Hashemite Kingdom of Jordan,” Mediterranean Politics 22 (2016): 464–83.

77 For more on the concept of political rationality, see Wendy Brown, Undoing the Demos: Neoliberalism’s Stealth Revolution (New York: Zone Books, 2015), 47–78.

78 Jillian Schwedler, Faith in Moderation: Islamist Parties in Jordan and Yemen (Cambridge: Cambridge University Press, 2006), 134; Luigi Achilli, “Becoming a Man in al-Wihdat: Masculine Performances in a Palestinian Refugee Camp in Jordan,” International Journal of Middle East Studies 47 (2015): 263–80.

79 His Majesty King of Jordan Abdullah, II, “Khitab al- ʿArsh al-Sami fi Iftitah al-Dawra al- ʿAdiyya al-Thaniyya li-Majlis al-Umma al-Sabʿat ʿAshir,” Amman, Jordan, 2 November 2014, accessed 4 August 2016, http://kingabdullah.jo/index.php/ar_JO/speeches/view/id/548/videoDisplay/0.html.

80 Senator Hani Mulqi, interview with the author, 4 May 2014, Amman.

81 Arabic 21,“Takhawafat Sha ʿbiyya wa-Hizbiyya min Raf ʿa Saʿar al-Khubz,” al-Sabeel, 14 May 2015, accessed 20 September 2015, .

82 The monarchy consistently appropriates policy measures that are seen to be “for the people.”

83 Shryock, “Dynastic Modernism,” 57–79.

84 Frisch, “Fuzzy Nationalism,” 86–103.

85 Michel Foucault, The History of Sexuality, Volume 1: Introduction, trans. Robert Hurley (New York: Penguin, 1990 [1976]), 100.

86 The dearth of Quʾranic references to bread contrasts quite clearly with their profusion in the hadith, in which it appears nearly twenty times. One of the meanings ascribed to bread does coincide with Saʿid’s remarks. Bread is mentioned on various occasions in al-Bukhari’s compilation as the appropriate means of almsgiving during the life of the Prophet Muhammad.

87 Many of Ahmad’s views are quite similar to the critiques of unbridled capitalism articulated by Jamal al-Din al-Afghani and Muhammad ʿAbduh. For more see Charles Tripp, Islam and the Moral Economy: The Challenge of Capitalism (Cambridge: Cambridge University Press, 2006), 33–34.

88 Nathan J. Brown, Arguing Islam after the Revival of Arab Politics (Oxford: Oxford University Press, 2016), 185.

89 Elena Corbett, “Hashemite Antiquity and Modernity: Iconography in Neoliberal Jordan,” Studies in Ethnicity and Nationalism 11 (2011): 163–93; Ahmad Khalif Al-ʿAfif, Qasim Muhammad Salih, and Muhammad Khalil al-Zabun, al-Tarbiya al-Wataniyya (Amman: Dar Jarir, 2013).

90 For an excellent example of this strand of discourse as it is articulated by King Abdullah II, see “Speech of His Majesty King Abdullah II at the Graduation of the 26th Class of Muta University’s Military Wing,” Amman, 16 June 2013. An English translation can be found at: http://kingabdullah.jo/index.php/en_US/ speeches/view/id/522/videoDisplay/0.html, accessed 20 November 2015.

91 For interesting historical parallels in rural Egypt, see Chalcraft, Popular Politics, 116–21.

92 Kevin J. O’Brien, “Rightful Resistance,” World Politics 49 (1996): 45.

93 Sherry B Ortner, “Resistance and the Problem of Ethnographic Refusal,” Comparative Studies in Society and History 37 (1995): 173–93.

94 Hamdi Barjus, bakery owner, interview with the author, 10 November 2013, Amman.

95 Tripp, Islam and the Moral Economy, 12; Deeb and Harb, Leisurely Islam, 25.

96 Wide variations in political views and policy preferences existed amongst bakers, which the BOA’s position does not and cannot fully capture.

97 Sayf al-Jenini, “al-Hamawi: Nataʾij Karithiyya li-Aliyya Daʿm al-Khubz wa-l-Naqid al-Mubashir Khi-yar Maqbul,”al-Raʾi, 23 January 2014, accessed 12 October 2014, http://www.alrai.com/article/628187.html.

98 Abdul Illah Hamawi, President of the BOA, interview with the author, 5 November 2013, Amman.

99 Many such targeting alternatives fail to account for the weak institutional capacity and corruption that plague social protection schemes in Jordan. For more on how new regimes of social payments may open up innovative political possibilities, see James Ferguson, Give a Man a Fish: Reflections on the New Politics of Distribution (Durham, N.C.: Duke University Press, 2015).

100 Sayf al-Jinini, “al-Hamawi: Nataʾij Karithiyya.”

101 O’Brien, “Rightful Resistance,” 31–55; Kevin J. O’Brien and Lianjiang Li, Rightful Resistance in Rural China (Cambridge: Cambridge University Press, 2006).

102 Baylouny, “Militarizing Welfare,” 277–303.

103 Yom, “The New Landscape of Jordanian Politics,” 284–300.

104 Asef Bayat, Life as Politics: How Ordinary People Change the Middle East (Palo Alto, Calif.: Stanford University Press, 2013).

105 Tariq Tell, “Early Spring in Jordan: The Revolt of the Military Veterans,” Carnegie Middle East Centre, 4 November 2015, http://carnegie-mec.org/2015/09/30/early-spring-in-jordan-revolt-of-military-veterans/iigv.

106 Ryan, “Political Opposition,” 367–90

107 Bohstedt, Politics of Provisions, 268.

108 Subsidized bread provision has also proven crucial to avoiding social unrest during wartime. For more, see José Ciro Martínez and Brent Eng, “Struggling to Perform the State: The Politics of Bread in the Syrian Civil War,” International Political Sociology 11 (2017): 130–47; and José Ciro Martínez and Brent Eng, “The Unintended Consequences of Emergency Food Aid: Neutrality, Sovereignty and Politics in the Syrian Civil War, 2012–15,” International Affairs 92 (2016): 153–73.

109 Scott, Weapons of the Weak, 337; Antonio Gramsci, Selections from the Prison Notebooks of Antonio Gramsci, ed. and trans. Quintin Hoare and Geoffrey Nowell Smith (New York: International Publishers, 1971), 181–82.

110 Frisch, “Fuzzy Nationalism,” 86–103.

111 I thank one of the anonymous reviewers for pushing me to emphasize this point.

112 Scott, “Afterword,” 399; José Ciro Martínez, “Jordan’s Self-Fulfilling Prophecy: The Production of Feeble Political Parties and the Perceived Perils of Democracy,” British Journal of Middle Eastern Studies 44 (2017): 356–72; Lisa Wedeen, Ambiguities of Domination: Politics, Rhetoric, and Symbols in Contemporary Syria (Chicago: University of Chicago Press, 1999).

113 Brian Steensland, “Cultural Categories and the American Welfare State: The Case of Guaranteed Income Policy,” American Journal of Sociology 111 (2006): 1273–326.

114 Arnold, “Rethinking Moral Economy,” 91.

115 Bohrstedt, Politics of Provisions, 262.

116 John Chalcraft, “Labour Protest and Hegemony in Egypt and the Arabian Peninsula,” in Social Movements in the Global South: Dispossession, Development and Resistance, ed. Sara C. Motta and Alf Gunvald Nilsen (New York: Palgrave Macmillan, 2011), 35–58.

